# A Brief Review on the High-Energy Electromagnetic Radiation-Shielding Materials Based on Polymer Nanocomposites

**DOI:** 10.3390/ijms22169079

**Published:** 2021-08-23

**Authors:** Angel Acevedo-Del-Castillo, Ernesto Águila-Toledo, Santiago Maldonado-Magnere, Héctor Aguilar-Bolados

**Affiliations:** 1 Facultad de Ciencias Químicas y Farmacéuticas, Universidad de Chile, Santiago 8380494, Chile; angel.acevedo@postqyf.uchile.cl (A.A.-D.-C.); ernesto.aguila@ug.uchile.cl (E.Á.-T.); santiago.maldonado@ug.uchile.cl (S.M.-M.); 2 Departamento de Polímeros, Facultad de Ciencias Químicas, Universidad de Concepción, Concepción 3349001, Chile

**Keywords:** polymeric nanocomposites, high-energy electromagnetic radiation, attenuation coefficient, high-energy electromagnetic radiation-shielding properties

## Abstract

This paper revises the use of polymer nanocomposites to attenuate high-energy electromagnetic radiation (HE-EMR), such as gamma radiation. As known, high-energy radiation produces drastic damage not only in facilities or electronic devices but also to life and the environment. Among the different approaches to attenuate the HE-EMR, we consider the use of compounds with a high atomic number (Z), such as lead, but as known, lead is toxic. Therefore, different works have considered low-toxicity post-transitional metal-based compounds, such as bismuth. Additionally, nanosized particles have shown higher performance to attenuate HE-EMR than those that are micro-sized. On the other hand, materials with π-conjugated systems can also play a role in spreading the energy of electrons ejected as a consequence of the interaction of HE-EMR with matter, preventing the ionization and bond scission of polymers. The different effects produced by the interactions of the matter with HE-EMR are revised. The increase of the shielding properties of lightweight, flexible, and versatile materials such as polymer-based materials can be a contribution for developing technologies to obtain more efficient materials for preventing the damage produced for the HE-EMR in different industries where it is found.

## 1. Introduction

High-energy radiation is produced by different phenomena; i.e., it comes from the decay of radionuclides or is irradiated by astronomical objects [1]. As is known, high-energy radiation is divided into two groups, emission particles, such as α alpha and β particles, neutrons, and high-energy photons. The latter are known as X-rays and gamma rays. Gamma rays correspond to the electromagnetic radiation of energies greater than 0.1 MeV. High-energy radiation produces drastic changes in matter by interacting with it. Therefore, they can cause considerable damage to electronic equipment and facilities [2,3]. However, the greater risk is that high-energy radiation can lead to carcinogenesis, cell mutation, or organ failure [4].

**Figure 1:**
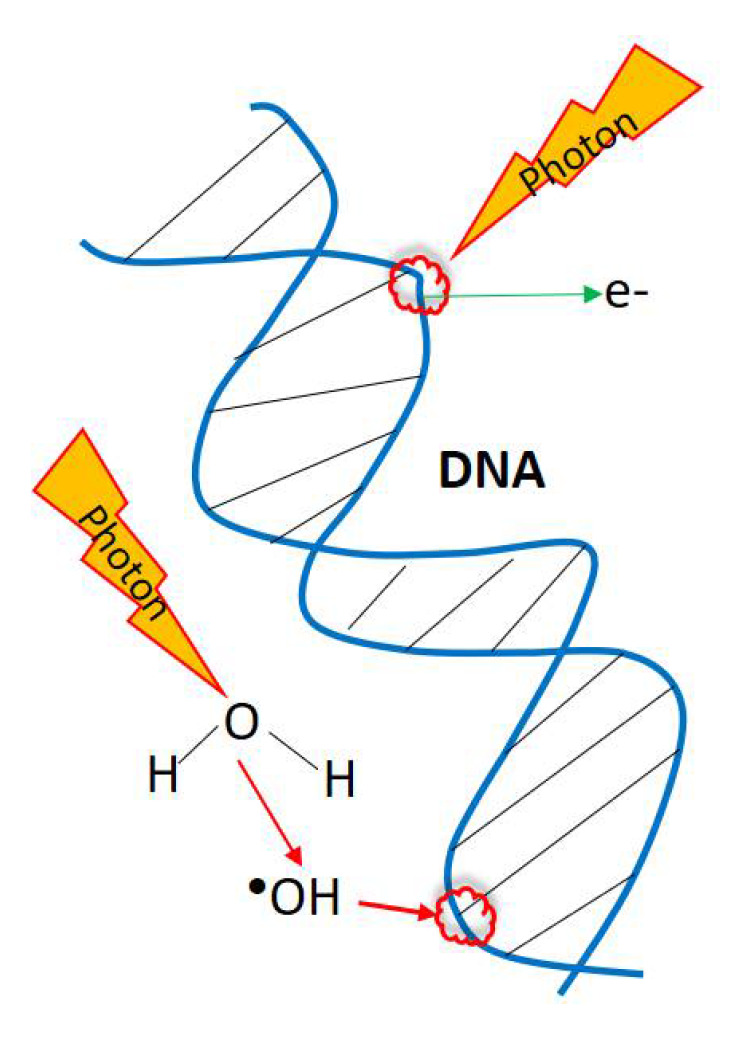
Scheme of the direct or undirect influence of the high-energy electromagnetic radiation on DNA.

Radiation damage in biological materials occurs when the radiation doses interact with their atoms, promoting the ionization. This ionization releases high energy and promotes the rupture of bonds of biological material such as DNA. This can occur directly or indirectly due to the generation of free radicals such as hydroxyl radical yielded from water present in cells (Figure 1) [5].

Therefore, protection against high-energy radiation is an important challenge to address. It is in this context that this review addresses new concepts in the development of new materials for radiation protection.

**Figure 2:**
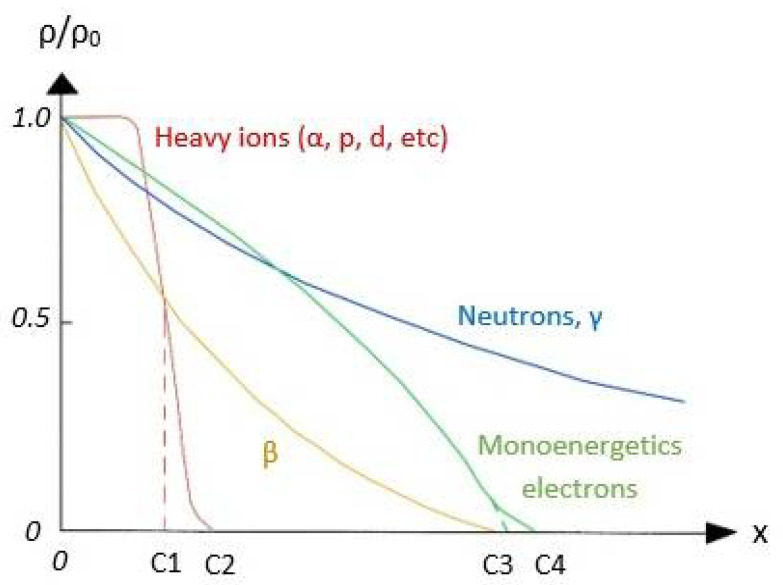
Curves showing relative transmission *ρ*/*ρ*_0_ as function of absorber thickness x. C1 and C3 are average, C2 and C4 are the maximum range. Reprinted from: Choppin G, Liljenzin J, Rydberg J. CHAPTER 6 Absorption of Nuclear Radiation, Radiochemistry and Nuclear Chemistry, Copyright (2002) with permission from Elsevier.

The emission particles, such as α and β, are of low penetration, although in their passage, they cause important damages in the matter. Figure 2 displays the relative transmission (*ρ*/*ρ*_0_) of a particles flux (*ρ*_0_) through and absorbing material with x thickness. The decrease in transmission is associated with the processes of interaction with matter. As seen, emission particles (α and β) are absorbed in average thickness ranges lower than neutrons or gamma rays. Moreover, alpha particles produce processes such as ionization, nuclear scattering, nuclear excitation, or nuclear transmutation. These processes involve collisions of the particles and consequently the emitted particles experience loss of energy. In addition, α and β particles are charged, so they are more susceptible to interact with the matter than uncharged particles, such as neutrons. On the other hand, it is observed in Table 1 that gamma radiation presents numerous processes when absorbed by matter, and its penetration into matter is only comparable with neutrons.

The sources of emission of these particles and high-energy electromagnetic radiation (HE-EMR) are present in several industries such as observational astronomy, the aerospace industry, radiological medicine, and nuclear industry [4]. It is important to notice that the aerospace industry demands materials that present enhanced mechanical and thermal properties, lightweight, and present a wide range of service temperatures. The latter is because the temperature in the stratosphere is in the range of −15 °C and 51 °C, while the temperature in outer space is ca. 2.7 K [6]. In this context, polymers are a viable alternative for the development of materials because several polymers present a wide service temperature range [7,8]. However, they lack resistance to high-energy radiation; consequently, they tend to be degraded with low radiation doses [9].

## 2. Interaction of Gamma Radiation with Matter

**Figure 3:**
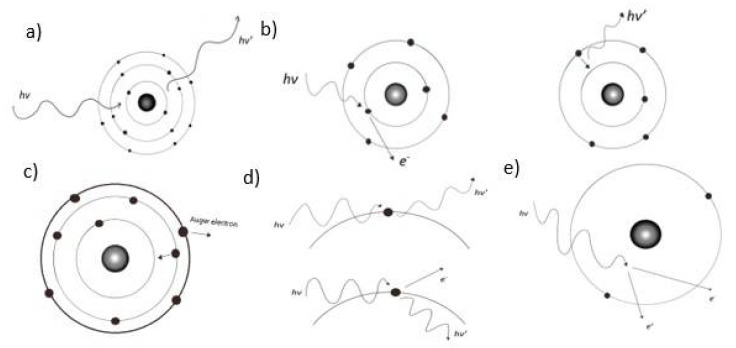
Schematic representation of Rayleigh scattering (**a**), the photoelectric effect (**b**), Auger effect (**c**), Compton scattering (**d**), and pair production (**e**).

To understand in a deeper way the processes that occur in the interaction, it is necessary to detail what constitutes the interaction processes of gamma radiation with matter. These processes are Rayleigh scattering, the photoelectric effect, Compton scattering, and pair production [10], which are schematically represented in Figure 3. These are briefly described below.

### 2.1. Coherent Scattering

The main interaction of matter with photons in the energy range between 100 and 2000 eV is coherent scattering [11,12]. Coherent scattering is an interaction with electrons belonging to an atom [13]. The energy transferred to the electron is too small to generate an excitation or ionization of the atom. Consequently, an elastic collision is generated, and the scattering of photons with the same wavelength of the primary radiation occurs. This scattering process is also called coherent, because the radiation scattered by each electron occurs in the same phase; therefore, a constructive interference of these occurs [14]. The probability that coherent scattering occurs increases by how much the value of Z increases, due to the increase in the number of electrons [14]. Coherent scattering plays a contributing role in the attenuation coefficient, but not significantly [15].

The atomic cross-section for coherent scattering and the mass attenuation coefficient decreases with increasing hν, where h is Planck’s constant and ν is the frequency of the electromagnetic wave [14,15,16].

### 2.2. Photoelectric Effect

The photoelectric effect is one of the ways of loss of energy of photons. This phenomenon consists of the absorption of an electromagnetic wave by an electron from the inner shells of an atom. The electron uses an amount of energy to break free from its strong bond with the atom and the rest is used as kinetic energy [17,18]. Consequently, the electron is ejected, and some electrons from more outer shells fill that vacancy, so an X-ray is emitted in the process. It can also happen that a second electron is ejected to remove excess energy. This electron is called the Auger electron [19]. The photoelectric effect strongly depends on Z, having dominance preferably in elements with high Z and with low energy photons. The greatest contribution of photoelectric absorption to the mass attenuation coefficient occurs at 88 keV [20].

### 2.3. Compton Scattering

This effect occurs when a photon hits an electron that is not bound and has a steady state, that is, a free electron. The energy of the photon is transferred to the electron that is emitted from the atom at a certain angle, which is called the Compton electron. Another photon with the remaining energy of the process, and therefore less than the incident radiation, is scattered at another angle [15,21]. This type of scattering differs from the coherent one by the interaction it performs only with an electron and not the atom. The energies of the scattered electron depend on the scattering angle [21].

The effect of the incident photon can continue to cause secondary ionizations because of scattered photons [16]. The scattered photons have enough energy to interact through the photoelectric effect, Compton, or pair production, generating other photons and Auger electrons [22].

### 2.4. Pair Production

Pair production consists of the formation of an electron and a positron from a photon, which disappears. This photon is transformed after passing through a Coulombic field, normally belonging to an atomic nucleus. This type of interaction of radiation with matter is the main one at high energies, above 1022 MeV [20,23]. This value is best described as Equation (1).

(1) $$E\geq 2m_{2}C^{2}$$

where *m_e_* is the mass of an electron and *c* is the speed of light. The minimum value of 1.022 MeV to produce pairs to occur is because it is the energy that an electron and a positron have in steady state (each with a value of 0.511 MeV) [21].

## 3. HE-EMR Attenuation

As commented, the interaction between high-energy radiation photons with matter implies the attenuation of this energy by the different phenomena, photo electronic effect, Compton process, or pair production. In this respect, the attenuation coefficient, *µ*, is defined as the capability of the absorbent, irradiated matter, to attenuate the energy radiation in a specific part of the EM radiation spectrum region, without presenting nuclear radiation. The radiation interaction with matter causes an exponential attenuation, as seen in Equation (2) [24]:

(2) $$I_{d}=I_{0}\;\cdot \;e^{-\mu d}$$

where *I_d_* is radiation intensity after attenuation, *I*_0_ is the incident radiation intensity, and d is the thickness of the absorbent material.

The energy loss of γ-rays, because of their interaction with matter, is attributed to the occurrence of the different interactions described in the previous section. It is important to notice that Equation (3) is only valid for a thin absorbent because it corresponds to a simplified equation, which does not consider the width of the absorbent. Moreover, if it is considered that Id corresponds the photon of photons transmitted per area unit from the matter after a radiation exposure of time t, the expression can be simplified and expressed in linear length units. Consequently, *μ* is referred to as the linear attenuation coefficient [22].

The attenuation coefficient can also be expressed depending on the density of the material (Equation (3)), which is a factor that has significant relevance in the interaction processes, because it allows considering the influence of the cross-section as well as the atomic number and mass number ratio (Z/A) [25].

(3) $$\mu_{m}=\frac{\mu }{\rho }\;$$

where *μ_m_* is called the mass attenuation coefficient and *ρ* is the density of the material [22].

As commented, the energy loss from incident radiation that travels through the absorbent material depends on several phenomena related mainly to the nature of the radiation. Particularly, the HE-EMR, such as gamma rays, can trigger different processes, such as elastic repulsions, inelastic coulombic interactions with the electron of the irradiated matter, or inelastic interactions with the nucleus, generating secondary particles [26]. Consequently, the attenuation coefficient should be understood as the contribution of the different phenomena (Equation (4)),

(4) $$\mu =\mu_{coh}+\mu_{phot}+\mu_{Comp}+\mu_{pair}$$

where subscript *coh*, *phot*, *comp*, and *pair* indicate the contribution of coherent scattering, photoelectric effect, compton effect, and pair production, respectively.

It shows the contribution of each of the partial absorption processes. Among the different parameters that have an influence, the dominant factor in the determined attenuation process is the energy of the incident beam.

It is important to mention that for a compound based on two or more elements, an average Z (atomic number) between the attenuating atoms is defined for understanding the attenuation of electromagnetic energy. This is the so-called effective atomic number Z_eff_ [27].

**Figure 4:**
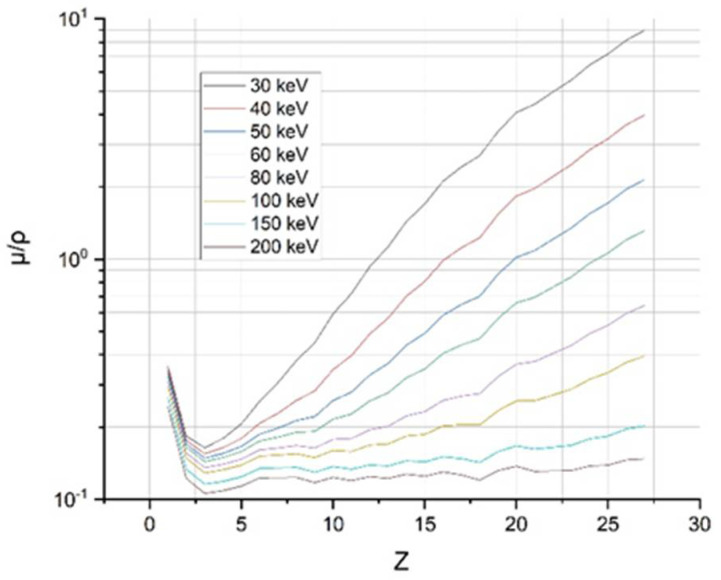
Mass attenuation coefficient *µ*/*ρ* (cm^2^/g) as a function of the Z of the first 26 elements for several energies.

Usually, when the attenuation coefficient is plotted as a function of the incident energy of some material, as in Figure 4, some sharp bands can be observed at certain energy values; this is due to the layers of K, L, M, etc. The signal from these layers for low Z atoms begins to appear below 20 keV. While for those elements with a high atomic number, these values are above 100 keV [27].

As seen in Figure 4, the attenuation coefficient increases with Z. Furthermore, it is possible to verify that at higher energies, the attenuation coefficient values are lower. The values of the attenuation coefficients for elements up to Z = 98 at different X-ray energy values are tabulated by the NIST (National Institute for Standards and Technology) [28]. In the same way, the growth behavior of the attenuation coefficient can be observed with the increase of the atomic number.

It is important to notice that other parameters have been proposed to evaluate the attenuation properties of the materials, such as the half value layer (*HVL*), tenth value layer (*TVL*), and mean free path (*MFP*), which are presented in Equations (5)–(7), respectively.

(5) HVL=0.693/μ

(6) TVL=2.303/μ

(7) MFP=1/μ

where *μ* is the linear attenuation coefficient. *HVL* corresponds to the absorbant thickness that reduces the incident radiation to 50%, *TVL* is the thickness required to reduce the incident radiation to 10%, and *MFP* is thickness that reduces the incident radiation to 36.8% [29].

### Prediction and Determination of Attenuation Coefficients

It is important to mention that by using the Boltzmann equation, the relationship between the interaction and transit of radiation through a material can be established. Consequently, it is possible to describe the balance of the flow of particles that pass through an infinitesimal volume of a body. An approach to solve this equation consists of considering specific characteristics of each system and resorting to computational simulation.

There are several types of software designed to model the attenuation capability of a specific material. The highlight among them is HZETRN, Geant4 used by the European Space Agency (ESA) [30] and CERN FLUKA, HETEC, PICARD, and EGSnrc [31]. Particularly, GEANT4 is based on a Monte Carlo method and provides different options to simulate different physics processes [25].

By using a Monte Carlo method for transport processes, it is possible to model a system where particles originate according to the distribution associated with the source and with an average free route that is the result of the probability distribution based on the total cross-section of the interaction, which eventually generates a collision or a change in the trajectory of the initial particle, giving rise to new particles that could interact in a similar way to the originals until it finally escapes the material [32].

To simulate a specific interaction process carried out using specialized software, the Monte Carlo method considers the use of number sequences that are called seeds. Consequently, pseudo-random numbers can be obtained [33]. The random number generator, on which a platform is based under this method, can contain about 10,171 numbers, where the variance (Equation (8)) represents the statistical uncertainty,

(8) $$S_{x}^{2}=\frac{1}{n-1}\overset{n}{\underset{i=1}{\sum }}{\left(x_{i}-x\right)}^{2}$$

where *n* corresponds to the batch amounts associated to the standard error. This simulation considers all the processes in which the particle can be involved along its journey through the mitigating material. The Monte Carlo method encloses a set of strategies to facilitate the manipulation of random numbers and thus simulate physical processes obtaining consistent results comparable with experimental results.

The validation of these results can be carried out by direct comparison with experimental results, using detectors suitable for the energy region of interest. Good adjustment between theoretical and experimental results has shown that simulation is an effective tool [34].

On the other hand, the response derived from the ionization and/or excitation of atoms and molecules when interacting with nuclear particles is the basis of individual detection based on a specific behavior. However, macroscopic features such as the heat differential can also be measured and related with the matter and radiation interaction process. Depending on this, different techniques have been developed for the quantification of nuclear particles that gives rise to detectors such as fog chamber, Geiger–Muller detector, SSNTD detectors, Cerenkov detectors, and Scintillator detector [35].

**Figure 5:**
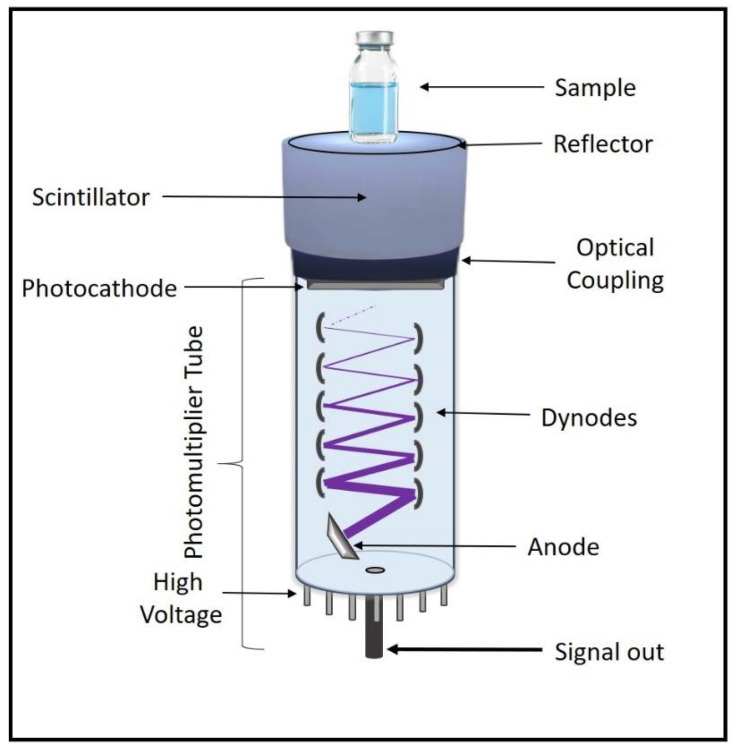
General diagram of the scintillator detector.

The most used detector to determine the γ radiation attenuation capacity of a material is the scintillator. In general, the scintillator detector is used to determine the transmitted energy from an absorbent located between the radiological source and the detector. As commented, the high-energy radiation produces multiple processes in matter such as the excitation of inner electrons, that among other relaxation processes can emit visible region photons, with proportional energy to incident radiation. The most commonly used scintillation detector is NaI(Tl) [36,37], showing excellent absolute efficiency. This detector corresponds to a type of inorganic scintillator, where the crystalline structure plays a fundamental role in the scintillation process. Electrons can occupy valence or conduction bands; in which they move freely through all crystals. There is a significant energy gap between the two levels. The relaxation process from the upper level by emission of a photon is unlikely, so an impurity is introduced (Tl in this case), called the activator, which modifies the crystal’s band structure, incorporating new energy levels in the previously banned band. Under these conditions, the rapid relaxation of electrons to lower levels by photon emission becomes possible. So, this is a useful scintillator due to possessing a high probability of absorption of the incident energy, having a high efficiency of emission of luminescence radiation after absorption, being transparent to its own radiations, and emitting into the spectral region to which the photomultiplier is sensitive [35]. Figure 5 shows a general scheme of such detectors. Here, we can see that the scintillator is attached to a photomultiplier through an optical coupling with a photoemissive window, allowing passage of the photon until the photocathode, where this signal multiplies around 10 times due to a series of dynodes. The dynodes’ potential is increasing to attract the electrons produced, which are located inside a vacuum glass tube; now, this electric current finishes on an anode and is translated through a processor.

It is important to mention that although the experimental determination is significant, Monte Carlo simulation allows predicting the process or degradation of matter across a large range of time. For instance, Tonguç Özdemir carried out a simulation approach to predict changes in the mechanical properties of bisphenol A-based polycarbonate (BPA-PC) composites filled with bismuth(III) oxide (Bi_2_O_3_) after periods of 15, 30, and 300 years [38]. This simulation was carried out using previous results [39]. The results of the Monte Carlo simulations show that the geometry, position, and type of radioactive waste placed inside the BPA-PC container will determine the dose distribution and how much will be encapsulated. Additionally, the storing radioactive sample at 19 cm between the sample and the walls of the container will not cause significant changes in the mechanical properties of the polymer. The increase in the concentration of (Bi_2_O_3_) as the filler of the BPA-PC decreases the dose absorbed by the polymer in a proportional way. The approach of the mentioned work is useful to understand the importance of Monte Carlo simulation and how it can help solve several issues related to the disposal of radioactive waste, design protective radiation devices, or estimate the lifetime of the specific materials in structures exposed to high-energy electromagnetic radiation doses.

## 4. Effect of HE-EMR on Polymers

Overall, carbon–carbon bonds are found in conventional thermoplastic polymers such as polyolefins. However, polymers consisting of heterochains present several different types of bonds, such as C-O, C-N, and C-S, among others. The effects of radiation on organic molecules are highly dependent on the molecular structure. The excitation energy is distributed throughout the entire macromolecule, so the rupture of the weakest bond is expected to occur. This process cand yield the formation of radicals, only if the excitation energy is greater than the bond energy. In this regard, the typical bond energy in organic compounds can provide information to the polymer resistance against radiation. For instance, the energies are ca. 3.9 eV, ≤6.4 eV, 8.4 eV, 10 eV, 3.5–4.5 eV, 3.7 eV, and 7.7 eV for C-C, C=C (aliphatic), C=C (aromatic), C≡C, C–H, C-O, and C=O bonds, respectively. The presence of conjugated π-bonds decreases the localization effect of the excitation energy at a specific chemical bond. This allows aromatic compounds to distribute the excitation energy throughout its structure, reducing the probability of de-excitation by bond dissociation and favoring other processes such as collision transfer. The most radiation-resistant organic compounds contain aromatic rings (polyphenylenes) and fused ring systems (naphthalene, etc.) [40].

The current challenge presented by high-tech industries such as aerospace, nuclear, or observational astronomy is associated with high-energy radiation. This type of radiation produces drastic changes in the chemical composition and physical properties of matter, damaging facilities, equipment, and instrumentation. In this context, an indispensable requirement in materials science is to study how this type of radiation affects the development of polymeric materials.

High-energy radiation is used for various purposes, such as initiator of polymerization reactions, grafting of side groups onto the main polymer chain, and the formation of cross-linking networks [41]. However, irradiation generates the induction of defects in polymeric materials that alter their electrical [42], mechanical [43], and chemical [44]. These changes are attributed to two phenomena: degradation by excision of the polymeric chain due to the breaking of covalent bonds or the crosslinking of chains due to the formation of free radicals that induce the generation of new chemical bonds [45]. On the other hand, polymers based on silicon showed that the perhydro poly siloxane possesses higher shielding properties in the range between 84 keV and 1.3 MeV compared with other siloxane-based polymers such as poly dimethyl siloxane [46].

### 4.1. HE-EMR-Induced-Crosslinking

**Figure 6:**
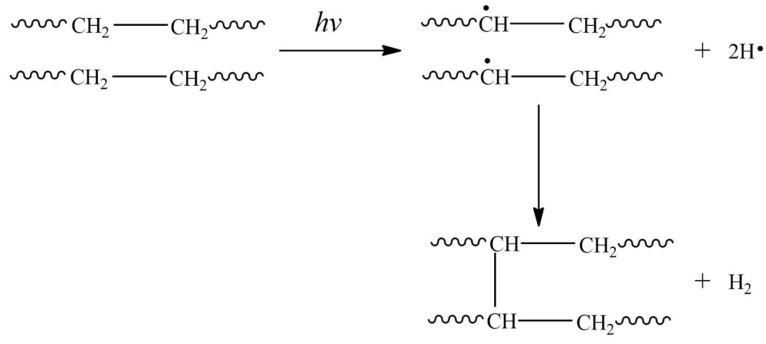
Representative scheme of the HE-EMR radiation crosslinking process.

Crosslinking is a dominant phenomenon in polymer irradiation because it generates new bonds in polymer chains, that is, the formation of intermolecular bonds (Figure 6). The first radiation cross-linked polymer was polyethylene [47]. Overall, crosslinking improves the mechanical and thermal characteristics of polymers in conjunction with chemical and environmental stability. During irradiation, both chain scission polymer degradation and crosslinking occur, but one of these phenomena may dominate depending on the dosage conditions [48]. Crosslinking depends on the radiation dose to which the polymer is exposed. Unsaturation or functional groups are not an indispensable requirement for the radiation-induced crosslinking process in the polymer, but polymers that contain aromatic groups in their structure are an exception to this fact. Currently, there is no general agreement on the exact nature of the crossover mechanism. The universally accepted mechanism is the cleavage of the C-H bond in a polymer chain to form a free radical, along with the release of atomic hydrogen. Subsequently, another hydrogen atom is abstracted by cleavage of the adjacent chain to form molecular hydrogen. The polymer chains generated by free radicals chemically react to form a new bond called a crosslinked bond. The crosslinking generates an increase in the molecular weight of the polymer and the formation of branched chains according to the radiation dose used. Finally, when all the free radicals of the polymer chains combine, a three-dimensional structure is obtained [49].

### 4.2. HE-EMR-Induced-Cleavage

**Figure 7:**
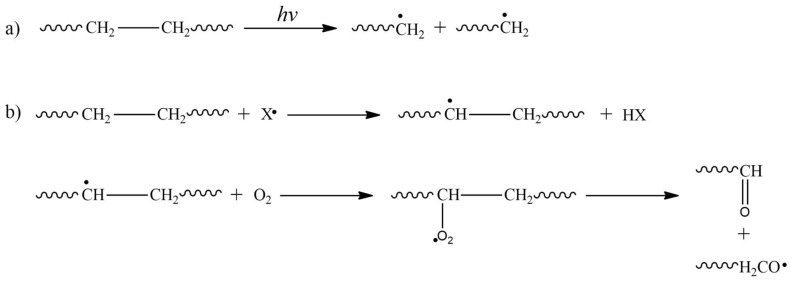
Representative schema of the HE-EMR-induced-cleavage. (**a**) C-C bond cleavage and (**b**) oxygen-induced cleavage, corresponding to an indirect degradation process.

The reverse process of crosslinking is the cleavage or degradation of the polymer chain (Figure 7). This phenomenon generates the reduction of molecular weight, together with a reduction of the mechanical properties such as elongation and the tensile strength of the polymer pristine.

The cleavage of the C-C bonds will occur whether the radiation is higher than the bonds’ energy (Figure 7a); otherwise, cleavage will take place in C-H bonds. Regarding the radiation effect in polymers in solution, this generates solvent free radicals, which promotes the generation of free radicals in the polymer chains (Figure 7b). In addition, the presence of oxygen in a solution and its interaction with polymer free radicals yields peroxides, which decompose in smaller molecules [49].

### 4.3. Specific Energy Requirement

Radiation-induced scission and crosslinking processes must be considered as radiation-induced chemical reactions, which require a specific energy to carry them out that is proportional to the adsorbed dose (*D*). It is defined as kilogray (kGy) as the dose equivalent to the absorption of 1 kJ per kilogram (kg) [50]:

(9) SE=D (kJ/kg)  (kGy).

*SE* is expressed in terms of the *G* value of the reaction, which is defined as the number of reactions for every 10^2^ electron volts (eV) of absorbed energy. Therefore, the specific energy is given by:

(10) $$SE=\;10^{2}(\mathrm{eV}/G)\times \;N_{A\;\;}\times \;1.602\;\times \;10^{\;-\;22}(kJ/eV)\;\times \;10^{3\;}/\left(kg\;mol^{\;-\;1}\right)\;$$

(11) $$SE=\left(9.65\;\times \;10^{6}/G\right)\;\left(kJ/kg\right)$$

where *N_A_* is Avogadro’s constant (6.022 × 10^23^ mol^−1^) and one electron volt (eV) is equal to 1.602 × 10^−22^ kiloJoules (kJ). The factor 10^3^/(kg mol^−1^) corresponds to correction for molecular weight.

By combining Equations (9) and (11), the expression for the dose is obtained [46].

(12) $$SE=D=\left(9.65\;\times \;10^{6}/G\right)\;\left(kGy\right)$$

Several reactions have ranges of G values between 0.1 and 10. The crosslinking G value of pristine polymers is typically 1. For instance, a polyethylene has a G value of 1 and a molecular weight of 100,000; a dose of 100 KGy is required to reach the maximum degree of crosslinking. In general, this dosage is sufficient for most industrial processes where a crosslinking process is required. On the contrary, if a polymer has high G values, a much higher dosage will be required, which is not feasible due to its high energy and processing cost. It has been reported that the scission process occurs in polymers such as polyisobutylene, polymethacrylates, polyvinylidene, and chloride polytetrafluoroethylene, among others, while the crosslinking process occurs in polymers such as polyethylene, polystyrene, polyacrylates, polyamides, and polyacrylamides.

The G values for crosslinking G (X) and for the scission G(S) chain in various pure polymeric materials are reported by Cleland et al. [50]. Materials with G (X)/G (S) ratios below 1 are preferable for applications where crosslinking is required. Polyethylene has a relationship of interest, since its G (S) values are approximately half of its G (X) values. Natural rubber has a very favorable crosslinking ratio due to its low G (S) value.

The G (X) and G (S) values can be modified by changing the dose. In general, it has been observed that the G (S) value increases with increasing the dose absorbed by the material compared to the G (X) value. Therefore, it is possible to control the values by controlling the level of applied dose. Therefore, polymers with a tendency to crosslink, such as natural rubber or typical polyethylene, can change to degradation by cleavage.

## 5. Composites as an Approach to Enhance the Performance of Polymers against HE-EMR

One difficulty in using polymers as high-energy radiation-shielding materials is their low durability when continuously irradiated. For instance, we used polyethylene (PE) and borated polyethylene (a mixture of PE and boron oxide) as neutron protection materials in nuclear reactors. These polymers lose mechanical and thermal stability when exposed to consecutive radiation [51,52,53,54]. Nevertheless, the advent of nanoscience and nanotechnology has fueled important advances in several fields, such as in medicine, electronics, and catalysts, among others. Likewise, nanoscience and nanotechnology have provided a new approach for the development of high-energy radiation-shielding materials [29,55,56,57,58,59,60,61]. For instance, different authors have reported the use of micro-sized and nanosized tungsten oxide as filler in diverse matrices such as epoxy or poly(vinyl chloride) composites in which nanosized structures showed a better shielding performance compared with micro-sized filler [62,63]. The nano and microparticles of lead(II) oxide have an effect on the gamma radiation-shielding and mechanical properties of nanocomposites based on an epoxy resin and unsaturated polyester [64]. The content of the microparticles was between 5.0 and 30 wt %, while the content of nanoparticles was between 1.0 and 5.0 wt %. The radiation sources were ^137^Cs (0.662 MeV) and ^60^Co (1.173 MeV and 1.332 MeV). The results of this study evidenced that the attenuation coefficient increased as the content of the filler increased. However, at similar content of micro and nanoparticles, the attenuation coefficients were higher for nanoparticles containing the nanoparticles than those observed in nanocomposites filled with microparticles. Additionally, the authors found that the increase of filler content enhanced the mechanical properties. Other research groups also have studied the effect of the lead-based materials on shielding properties on a variety of polymers and blends. In this regard, Pb_2_O_3_ nanoparticles were dispersed in polyvinyl ester as a matrix and irradiated to an electron beam of 50 kGy to crosslink the polymer and obtain nanocomposites with enhanced attenuation properties. The attenuation percentage of the resulting nanocomposite was 86% compared to that of the neat polyvinyl ester, which was 32% [29]. Poly(vinyl acetate) containing up to 30 wt % of Pb(NO_3_)_2_ also evidenced an increment of the attenuation coefficient [65]. Similarly, in a polymer blend based on carboxymethyl cellulose (CMC)–polyvinyl pyrrolidone (PVP) reinforced with lead(IV) oxide nanoparticles, a high increment of the attenuation coefficient was observed [66]. Nanocomposites containing lead not only present shielding properties to gamma radiation but can also protect against neutrons. For instance, the mass attenuation values were higher to low values of radiative energy (10 keV and 0.8 MeV) of natural fiber high-density polyethylene and lead oxide composites [67]. Lead-based materials have shown more efficient shielding properties against gamma radiation; for instance, polymeric nanocomposites based on epoxy resin with inorganic fillers, such as Pb, Zn, ZnO, Ti, and TiO_2_ were studied, and the results showed that these composites based on epoxy and Pb 25 wt % presented the highest shielding properties [68].

Concerns related to the use of lead in high-radiation protective materials have grown due to the health risk associated with this heavy metal [69]. Different studies have been oriented to the development of lead-free radiation-shielding materials using Bi_2_O_3_, WO_3_, and MoO_3_ [70,71]. Likewise, lead-free natural fillers such as Yahyali Sotone, which is composed by Fe_2_O_3_ (75.28 wt %), SiO_2_ (17.21 wt %), and Al_2_O_3_ (4.24 wt %), have been used to obtain epoxy resin nanocomposites. It was observed that the composites showed radiation-shielding properties for low-energy radiation [72].

Recently, different authors have studied the influence of Bi-based compounds in polymers such as unsaturated polyester or polyimide, obtaining highly protective characteristics against γ-radiation [66,67]. For instance, Abdalsalam et al. [61] prepared and characterized nanocomposites for the shielding of high-energy radiation based on high molecular weight polyethylene filled with 0.5, 1, 1.5, and 2.0 wt % of bismuth(III) oxide. The radiation source was of ^133^Ba in the energy range between 30.8 and 383.9 KeV. For the lower incident energies, the Bi_2_O_3_ content has a high influence on the attenuation, which decreases as the energy of incident radiation increases. Likely, bismuth(III) oxide/high-density polyethylene-based nanocomposites have shown satisfying results in attenuation properties [61,73]. Other matrices such as poly(vinyl alcohol), polyimide, or isophthalic resins have been used for the preparation of Bi_2_O_3_-based composites. All of them have shown increment in their attenuation properties, especially when the filler corresponds to nanoparticles [74,75]. Moreover, composites material consisting of silicone rubber (poly-dimethylsiloxane) (PDMS) and bismuth (III) oxide was developed as flexible, non-toxic, and X/gamma ray shielding material. The attenuation rate was of 96.4% [76]. Nanocomposites based on high-density polyethylene using nano-Bi_2_O_3_ as fillers in gamma radiation attenuation properties also was studied. These composites were prepared by a hot pressing technique using filler concentrations between 0.5% and 2.0%. The dose range studied is between 30.82 and 383.9 KeV. The maximum mass attenuation coefficient observed under these conditions was obtained using 2.0% nano bismuth (III) oxide [73]. Polycarbonate-based nanocomposites containing bismuth (III) oxide amount up to 50 wt % were prepared by using a solution mixing method. The attenuation coefficients of these nanocomposites were determined using a CsI(Tl) detector and ^241^Am, ^57^Co, ^99m^Tc, and ^133^Ba as gamma rays sources. The results shown that the attenuation coefficients were increased significantly. Namely, the nanocomposite containing 50 wt % of bismuth oxide nanoparticles showed an increase of twenty-three times the attenuation coefficient compared with the pristine PC [77].

Furthermore, Ambika et al. [74] obtained results where the composites based on unsaturated polyester and Bi_2_O_3_ presented comparable performance to that using baryte (BaSO_4_) as filler. One aspect that could result in a disadvantage for the use of Bi_2_O_3_ as filler in polymers is the relatively low thermal conductivity of Bi_2_O_3_ (3.53 Wm^−1^K^−1^) compared with that of lead, which is ten times higher (35.3 Wm^−1^K^−1^) [78]. A comparative study by Monte Carlo simulation has shown that the combination of PbO and Bi_2_O_3_ nanoparticles as filler in silicone-based composites has shown higher performance than those composites based on silicone and WO_3_.

Other alternatives to replace lead-based filler also have been studied. For instance, the radiation-shielding, optical, and structural properties of PVA films containing BaTiO_3_ nanoparticles were investigated. ^133^Ba, ^152^Eu, and ^137^Cs were used as sources for measuring the shielding properties of the nanocomposites. The radiation transmission decreases, and reflectance increases as the BaTiO_3_ content in the nanocomposites was increased [65]. The gamma radiation-shielding properties were determined for nanocomposites based on unsaturated polyester and Nb. Composites containing 15 wt % of Nb not only showed the highest radiation protection efficiency but also showed the highest absorbent power ratio [79]. Likewise, nanocomposites based on low-density polyethylene containing variable amounts of W nano and micro-sized particles were prepared, and we evaluated their relative reduction of transmission (RRT). The authors found that the micro-sized nanoparticles were more effective at reducing the transmission, and this reduction was proportional to the filler content [80]. Composites based on polyether ether ketone prepared by 3D printing also have been studied; these composites showed increased shielding efficiency against the radiation emitted by ^60^Co and ^137^Cs sources with content of tungsten in the range between 50 and 70 wt % [81].

To obtain a summative or synergistic effect on the attenuation properties, different studies have been carried out considering the combination of fillers. In this regard, it has been reported that ternary nanocomposites based on polypropylene CdO and bentonite presented an increase of the HVL and mass attenuation coefficients to gamma radiation [82]. In the case of silicone elastomers, the preparation of silicone rubber composites based on (polydimethylsiloxane), bismuth (III) oxide, and hexagonal boron nitride (hBN) with shielding properties against neutron, X, and gamma radiations was reported [83]. The shielding properties of poly (vinyl butyral) containing Bi_2_O_3_@BaZrO_3_ also have been studied, and it was observed that they were significantly increased [84]. Moreover, ternary composites based on polyetherimide containing hexagonal boron nitride and gadolinium oxide (Gd_2_O_3_) prepared by the melt-mixing process were reported. The mass attenuation coefficient for gamma radiation was slightly increased, which was attributed to the low content of the fillers and their low atomic number [85]. However, the use of a binary filler system does not always produce the expected increment of the shielding properties; those fillers with higher Z will have a more dominant effect on the radiation attenuation [86].

The development of several carbon-based nanomaterials in recent decades such as carbon materials and carbon nanotubes, among others, has fueled the scientific research approach to the development of electrically and thermally conducting polymers nanocomposites [87,88,89]. In addition, the use of carbon-based nanomaterials also contributes to obtain nanocomposites with enhanced mechanical properties. Particularly, graphene is a polymorph of carbon in which the atoms are exclusively linked by sp^2^ bonds. As a result, these carbon atoms are arranged in a long-range hexagonal lattice of monatomic thickness, which presents a long-range π-conjugated system [90]. Materials that are structurally similar to graphene are denominated graphene materials, which is a family of materials that has grown rapidly [91].

As known, π-conjugated systems present high resistance to high-energy radiation, because they can spread the energy over the whole system, favoring the de-excitation of electrons by collisional transfer rather than by dissociation. Therefore, condensed ring systems such as naphthalene are the most resistant organic compounds to high-energy radiation [40]. This indicates that the graphene content homogeneously dispersed into the polymer matrices can spread the energy of photoelectrons produced by the interaction of high-energy radiation with the nanocomposite, promoting their recombination with their geminate (i.e., original ion pair).

Among the graphene-based nanomaterials, few-layer graphene, graphene quantum dots, graphene nanoribbons, graphene oxides, functionalized graphene, reduced graphene oxide, and doped graphene are found [92]. The scientific community has shown interest in graphene materials due to their outstanding characteristics, which will allow developing several applications in diverse technological fields. Electrical conductivity, as high as those exhibited by metals, and thermal conductivity, higher than that shown by diamond or single-walled carbon nanotubes, are some of these outstanding characteristics. Moreover, theoretical and experimental studies have recently reported an ultrafast thermalization of electrons because of their interactions with the graphene lattice [93,94,95]. However, although graphene was discovered in 2004 and intensely studied since that date [96], the challenges that are involved in developing large-scale production methods of graphene materials have relatively limited its technological use. Among the several methods reported in the literature for obtaining graphene materials, reduction of graphite oxide is considered as one of the most viable and easy-to-scale methods. This procedure enables obtaining graphene material of few-stacked layers, low oxygen content, and large surface area.

The approach to the production of reduced graphene oxide consists in the thermal or chemical reduction processes of graphite oxide promoting the exfoliation of stacked graphene layers. The graphite oxide can be obtained by several methods from graphite; however, Brodie’s and Hummers’ methods are the most used for its production [97,98,99,100]. As mentioned, different researchers have addressed the use of graphene materials as filler in polymer nanocomposites [101,102,103,104,105,106,107,108,109,110], since these nanocomposites can show high electrical conductivity (σ), achieving values compared to semiconducting materials. These nanocomposites with enhanced electrical conductivity have different applications. For instance, it has been reported that composites with σ101 Sm^−1^ can be used in the development of materials for electromagnetic interference (EMI) shielding [88]. Consequently, the addition of graphene materials to conventional or functional polymers can broaden drastically the range of applications of these polymers [109,111,112,113].

The homogeneous dispersion of graphene into the matrix will increase the efficiency of the transport properties. Such properties will be imparted by the long-range π-conjugated system of graphene layers, which additionally can play a role for preventing the degradation of the polymer matrix produced by high-energy radiation. The interest of using graphene-based polymer composites as shielding for high-energy radiation has grown. For instance, Viegas et al. [114] reported that graphene oxide/PVDF composites present an attenuation coefficient four times with respect to that observed for graphite/PVDF. Likewise, Hashemi et al. [115] reported that lead oxide-decorated graphene oxide/epoxy composites presented an X-radiation attenuation coefficient increase of up to 124.6% compared to neat epoxy matrix. On the other hand, although graphene or graphite-based polymer nanocomposites present increased attenuation coefficients compared with the attenuation coefficients of neat polymer matrices or the presence of these types of filler can spread the energy of photoelectrons, elevated doses of high-energy radiation will produce changes in the structure of materials as well as in their mechanical, electrical, and thermal properties. It has been reported that γ-radiation strongly affects the lattice of few-layered graphene materials [116]. Changes in the lattice of graphene materials can modify their heat and charge transport properties. Therefore, the monitoring of thermal and electrical conductivity of graphene-based nanocomposites could provide information about the degree of damage of lattices of graphene material. This in turn can give information about the effectiveness of a graphene lattice for the de-excitation of escaping electrons produced in a photoelectric effect or Compton scattering, resulting from the γ-radiation interaction with nanocomposites. It is interesting to note that Xie et al. found that composites based on graphdiyne and albumin exhibited a high free radical scavenging activity for the radiation protection of cells [117].

It is important to mention that the gamma radiation can be useful to obtain more resistant materials [118], this approach consists of using specific dose gamma radiation, which can favor increments in crosslinking, crystallinity, and adhesion between the filler and polymer matrix. For instance, the tensile strength of EPDM/clay nanocomposites was increased because the irradiation, which was attributed to the crosslinking induced by the radiation doses, and the irradiated nanocomposites exhibited higher resistance to the radiation than those unirradiated nanocomposites [119]. A similar effect on the mechanical properties of the radiation was observed in EPDM-CIIR nanocomposite blends that were prepared using MWCNT fillers and hybrid filler systems of MWCNT/nano clays. The results showed that the use of MWCNT reduces the formation of free radicals produced by gamma radiation. Furthermore, the use of the hybrid mixture confers superior resistance against gamma radiation to the nanocomposite due to the increased tortuosity in radical migration attributed to the presence of the nano clay [120]. Thermal properties also can be enhanced by the irradiation of composites [121,122]. In this regard, it was reported to irradiate carboxylated poly(vinyl chloride) nanocomposites containing silver or palladium nanocomposites to doses between 25 and 150 kGy to enhance the thermal stability and increase the thermal decomposition activation energy of the amorphous phase of the polymer [123]. As mentioned, the crystallinity of the polymer can be affected by the gamma radiation doses. It was found that the crystallinity and the crystal size of polyethylene terephthalate were increased as the dosage was increased. This was attributed to the break of the lamellas into two or more portions and the rupture of the amorphous region chains producing shorter chains, which can move freely to order themselves in a stable position into a regular arrangement [124]. Similarly, researchers observed an increase of the crystallinity in irradiated lignocellulosic composites based on polypropylene; concomitantly, an increase of the adhesion between the filler and polymer matrix was observed [125]. Regarding the charge transport properties, the exposition of the nanocomposites to the radiation enhances the electrical conductivity [126,127] and ionic mobility [128].

## 6. Outlooks and Future Trends in Materials for Protection against HE-EMR

In polymers technology, the use of a hybrid filler system enables the combination of two materials of different nature to impart specific properties to the resulting composites [109,129,130,131,132,133,134]. In this regard, the use of a hybrid filler system based on carbon-based nanomaterials and high-Z-based nanomaterials is seen as a viable and novel approach for obtaining polymeric nanocomposites for advanced applications. It is possible to expect a contributive effect. For instance, carbon-based nanomaterials that have a π-conjugated system can play a role to spread the energy of electrons ejected from atoms and prevent the bonds scission, while the high Z of post-transitional metals such as bismuth or lead-based compounds increases the attenuation properties of composites. Thus, there are interesting alternatives for manufacturing more resistant and lightweight materials based on polymer composites. However, different aspects should be considered such as the dispersion, size of nanoparticles, and interaction particles with polymers. These nanocomposites can find applications in different areas, such as aerospace and observational astronomy, among others.

## 7. Conclusions

There are several aspects that should be considered for developing gamma radiation-shielding materials based on polymers. The most relevant are to know the behavior of the polymers under gamma irradiation and their attenuation properties. The addition of materials based on elements with high atomic number will help to increase the attenuation properties, and these properties will increase proportionally to the content of the fillers. However, it is important to consider the toxicity of these fillers. In this regard, currently, lead-free fillers, such as bismuth oxide, are preferred for use in polymers for the development of the gamma-radiation-shielding polymeric materials. By combining different types of fillers, a summative effect on the attenuation properties can be observed. Moreover, the structure of the filler can help prevent the degradation of polymers. For instance, layered materials such as clays hinder the migration of radicals yielded by the interaction of the high electromagnetic radiation with the polymers. Likewise, materials that present a π-conjugated system, such as graphene, can help to spread the energy of electrons ejected from atoms because of the gamma radiation.

## Figures and Tables

**Table 1:** Nuclear gamma radiation absorption processes.

Photons (γ) Reacts with	Type of Reaction	Name of Process
Field of orbital electrons	γ scattered without energy loss	Coherent scattering
Free (outer) electrons	γ scattered with energy loss, ionization	Compton effect
Bound (inner) electrons	γ completely absorbed, one electron knocked out	Photo effect
Field of nuclear force	γ annihilated, formation of positron–negatron pair (E > 1.02 MeV)	Pair production
Atomic nucleus	γ scattered without energy loss	Mössbauer effect
	γ scattered with energy loss	Nuclear excitation
	γ absorbed by nucleus, nuclear transmutation (E > 5 MeV)	Nuclear photo effect

## Data Availability

Not applicable.
